# Supplementary material for: A systematic approach to study the effects of acquisition parameters and biological factors on computerized mammography analysis using ex vivo human tissue: A protocol description
Source: PLoS One. 2025 Aug 18;20(8):e0321658. doi: 10.1371/journal.pone.0321658 (PMC12360507; doi:10.1371/journal.pone.0321658)
Supplement: Attachment — Submitted filename: Response letter.pdf [file pone.0321658.s002.pdf]

Response letter

Reviewers' comments:

Comments to the Author

1. Does the manuscript provide a valid rationale for the proposed study, with clearly identified and justified research questions?

The research question outlined is expected to address a valid academic problem or topic and contribute to the base of knowledge in the field.

Reviewer #1: Yes

Reviewer #2: Yes

> Our response: We thank the reviewers for their initial comments that greatly helped us improve our manuscript.

2. Is the protocol technically sound and planned in a manner that will lead to a meaningful outcome and allow testing the stated hypotheses?

The manuscript should describe the methods in sufficient detail to prevent undisclosed flexibility in the experimental procedure or analysis pipeline, including sufficient outcome-neutral conditions (e.g. necessary controls, absence of floor or ceiling effects) to test the proposed hypotheses and a statistical power analysis where applicable. As there may be aspects of the methodology and analysis which can only be refined once the work is undertaken, authors should outline potential assumptions and explicitly describe what aspects of the proposed analyses, if any, are exploratory.

Reviewer #1: Yes

Reviewer #2: Yes

> Our response: We thank the reviewers for their initial comments that greatly helped us improve this section of the manuscript.

3. Is the methodology feasible and described in sufficient detail to allow the work to be replicable?

Descriptions of methods and materials in the protocol should be reported in sufficient detail for another researcher to reproduce all experiments and analyses. The protocol should describe the appropriate controls, sample size calculations, and replication needed to ensure that the data are robust and reproducible.

Reviewer #1: Yes

Reviewer #2: Yes

> Our response: We thank the reviewers for their initial comments that greatly helped us improve this section of the manuscript.

4. Have the authors described where all data underlying the findings will be made available when the study is complete?

The PLOS Data policy requires authors to make all data underlying the findings described in their manuscript fully available without restriction, with rare exception, at the time of publication. The data should be provided as part of the manuscript or its supporting information, or deposited to a public repository. For example, in addition to summary statistics, the data points behind means, medians and variance measures should be available. If there are restrictions on publicly sharing data—e.g. participant privacy or use of data from a third party—those must be specified.

Reviewer #1: Yes

Reviewer #2: Yes

Our response: We thank the reviewers for their initial comments that greatly helped us improve our manuscript.

5. Is the manuscript presented in an intelligible fashion and written in standard English?

PLOS ONE does not copyedit accepted manuscripts, so the language in submitted articles must be clear, correct, and unambiguous. Any typographical or grammatical errors should be corrected at revision, so please note any specific errors here.

Reviewer #1: Yes

Reviewer #2: Yes

Our response: We note that the language used is satisfactory.

6. Review Comments to the Author

Please use the space provided to explain your answers to the questions above and, if applicable, provide comments about issues authors must address before this protocol can be accepted for publication. You may also include additional comments for the author, including concerns about research or publication ethics.

You may also provide optional suggestions and comments to authors that they might find helpful in planning their study.

(Please upload your review as an attachment if it exceeds 20,000 characters)

Reviewer #1: I congratulate the authors since they have addressed all of the remaining issues. My suggestion is that the authors report the thickness of the tissues during imaging.

Reviewer #2: Thank you for your careful edits, I think that they clarify and answer all the issues I raised in my earlier review.

Our response: We thank the reviewers for their comments that greatly helped us improve our manuscript, and we are happy that the actions taken appropriately addressed their points.

Staff editor message:

Dear Dr. Hernández,

I am writing with an update about your submission, "A systematic approach to study the effects of acquisition parameters and biological factors on computerized mammography analysis using ex vivo human tissue: A protocol description" (PONE-D-24-30561R1).

During the submission process] you requested several authorship changes.

The nature, extent and timing of the requests to change the author list call into question whether your manuscript complies with the PLOS Authorship policy (https://journals.plos.org/plosone/s/authorship). As such, we are rescinding the Accept decision and rejecting the manuscript.

We may reconsider this submission in the future if a research integrity official at the corresponding author's institution reviews and provides verification of the article's authors and their contributions. The following documents would need to be provided when resubmitting:

a. Written, signed statements from all contributors, including added/removed authors, confirming that all agree with the article's author list and contributions

b. Cover letter that describes the contribution of each author and provides a specific reason why each author was added or removed after initial submission

c. Formal letter from a research integrity official or equivalent at the corresponding author's institution, or the institution where the majority of the research was conducted, confirming the author list and stated contributions.

d. Institutional email address for the official responsible for oversight of research and/or research integrity at the corresponding author's institution.

I am sorry we do not have more positive news, but hope that you understand the reasons why we rejected this submission.

Kind regards,

Joanna Tindall, PhD

Staff Editor

PLOS ONE

Our response: We disagree with the claims of this letter, specifically the claims stating, "*During the submission process] you requested several authorship changes*" and "*The nature, extent and timing of the requests to change the author list call into question whether your manuscript complies with the PLOS Authorship policy. As such, we are rescinding the Accept decision and rejecting the manuscript.*" We find the first statement untrue, as we requested one change, and find no basis for the second statement, as the nature of this request, that we outlined in the Request for Change to Authorship form - that we previously submitted to the journal - is compliant with the authorship policy, we submitted the requested written agreement from all authors, the extent is minimal as it is just one author, and the timing occurred at the only reasonable time where this could have occurred – when addressing the reviewers' initial comments and preparing the revised manuscript.

Given this, we believe the rejection of the manuscript was based on incorrect claims, and reassessment is warranted.

Revisions carried out on the manuscript

This manuscript had undergone one round of revision since its original submission. All reviewer comments were addressed appropriately, as reflected in the reviewers' subsequent feedback, shown at the beginning of this document. Additionally, in accordance with the journal's requests, we have removed the Competing Interests Statement, Authors' Contributions, and Data Availability sections from the main body of the manuscript and transferred them to the designated fields within the submission system.

The modifications mentioned can be identified in the Track changes manuscript file.
