# Supplementary material for: A systematic approach to study the effects of acquisition parameters and biological factors on computerized mammography analysis using ex vivo human tissue: A protocol description
Source: PLoS One. 2025 Aug 18;20(8):e0321658. doi: 10.1371/journal.pone.0321658 (PMC12360507; doi:10.1371/journal.pone.0321658)
Supplement: Attachment — Submitted filename: response_to_reviewers.docx [file pone.0321658.s001.docx]

November 13, 2024

Tampere, Finland

Editor-in-Chief

PLOS One

**Dear Editor-in-Chief**

Please find attached our revised manuscript titled “A systematic approach to study the effects of acquisition parameters and biological factors on computerized mammography analysis using ex vivo human tissue: a protocol description” (manuscript number: PONE-D-24-30561).

We are grateful for being allowed to revise our manuscript. Our manuscript received insightful and constructive suggestions. We incorporated these suggestions and believe the manuscript has improved significantly. Most notably, we have added necessary clarifications and details to the methodology. Please find our point-by-point responses below. The undertaken changes are highlighted in the tracked manuscript.

We look forward to hearing from you.

Sincerely, on behalf of all the authors,

Nicole Hernández, M.Sc.

Faculty of Medicine and Health Technology

Tampere University

**Point-by-point response letter**

**Reviewer 1:**

The authors proposed a protocol to collect a new dataset for investigating the effects of biological and non-biological heterogeneity on the mammograms. The research idea is original and reasonable. In general, the manuscript is carefully written. The aims of the protocol were explained in detail. Here are my suggestions:

Thank you very much for your comments. We address your suggestions below.

1) Figure 1 is a little bit misleading. The biological factors should be on top and the arrows should be directed from the factors to the mammographic texture features. In addition, imaging parameters should be given as a list separately. An arrow should be directed from the list to the mammographic texture features. This may be more informative for the readers.

Thank you for the suggestion. We agree the suggestion improves the Figure.

Actions taken: We have modified the figure accordingly. The figure is presented below.

2) The authors should explain why they used “dense” in line 171.

Actions taken: We have replaced the term “dense” by a more descriptive term “multi-parametric” to illustrate one of the main characteristics of our prospective dataset.

We wrote earlier: “Our study will produce a dense imaging dataset of mammograms.” The now write: “Our study will produce a multiparametric imaging dataset of mammograms.”

3) In Figure 3, the “man symbol” may be replaced by a “woman symbol” since most of the participants will be women.

Thank you for the suggestion.

Actions taken: We have modified the figure accordingly. The figure is presented below.

**Reviewer 2:**

This is an important study on a very interesting subject matter. I think however that the background and aims are hard to follow, and the study as a whole could benefit from a more focused narrative and more clearly explained purpose.

Thank you very much for your comments. We address your suggestions below.

BACKGROUND

Line 21-22: Calling breast cancer one of the most lethal cancers world wide is true in an absolute population sense, but relatively speaking and for the individual it is certainly not among the most lethal with ~95% five-year survival. Please rephrase.

Thank you very much for this important comment.

Actions taken: We have added the following information about the survival rate of breast cancer to subsection *1.1 Mammographic imaging and the current use of artificial intelligence.*

Early detection is of key importance for reducing breast cancer-related mortality [1, 2], as these cases have a 5-year survival probability of approximately 96% [3].

Line 22-29: It would be helpful for readers that are less familiar with breast cancer and mammography if you could provide some numbers backing up your points here, e.g. how much does screening reduced mortality, what is the percentage of breasts that are considered dense, how much does the risk depend on density and what is the sensitivity in various density groups.

Thank you very much for this insightful suggestion. We agree that the manuscript would benefit from more in-depth introduction.

Actions taken: To address the suggestion, we have amended the Introduction chapter.

We wrote earlier: Breast cancer is the most diagnosed and one of the most lethal cancers among females globally; there were 2.3 million new cases and 685,000 deaths in 2020 [1]. Mammographic breast cancer screening is currently used to detect breast lesions and plays a significant role in early cancer detection [2], which is of key importance for reducing breast cancer-related mortality [3, 4] Mammography screening is, however, less effective on breasts with higher percentage of fibroglandular tissue (FGT) - i.e. mammographically dense breasts -, due to the similarity in the radiodensity between FGT and breast tumor tissue leading to reduced contrast between these tissues [5, 6]. Furthermore, research has shown that higher breast density is associated with a greater risk of breast cancer within the screening population [7, 8]. Indeed, mammographic breast cancer screening is less effective in females with mammographically dense breasts who simultaneously have the highest risk of breast cancer.

We write now: With 2.3 million new cases and 685,000 deaths in 2020, breast cancer is the most diagnosed cancer and accounts for most cancer deaths among females globally [4]. Early detection is of key importance for reducing breast cancer-related mortality [1, 2], as these cases have a 5-year survival probability of approximately 96% [3]. Mammography screening programs, in which females aged 50 to 69 are invited for a biennial mammogram, are implemented across the EU [5]. Screening plays a significant role in early detection of the disease [6], as it has been shown that the successful implementation of screening programs can reduce breast cancer mortality by approximately 20% [7]. Mammography screening, however, is less effective on breasts with a higher percentage of fibroglandular tissue (FGT)—i.e., mammographically dense breasts—due to the similarity in the radiodensity between FGT and breast tumor tissue leading to reduced contrast between these tissue types [8, 9]. The sensitivity of breast cancer detection decreases with increasing breast density, from 85.7% in the lowest density category to 61.0% in the highest density category [10, 11]. Mammographically dense breasts (either heterogeneously dense or extremely dense) are found in about 48% of the screened population [12], and research has shown that females with higher breast density have a greater risk of developing breast cancer [13, 14]. Indeed, mammographic breast cancer screening is less effective in females with mammographically dense breasts who simultaneously have the highest risk of developing breast cancer.

Line 32-39: This definition of radiomics is not one I am familiar with. Radiomics is normally either a set of specific standardized quantitative features of radiological images that have been found to correlate with various genomic factors, or sometimes any quantitative image features when used in this manner. Radiomics has no direct relationship to machine learning, and indeed one could call the entire idea of using AI-analysis of images to find biomarkers as simply AI-driven radiomics.

Thank you. We agree with the corrections pointed out by the reviewer.

Actions taken: We have modified this section.

We wrote earlier: The classical machine learning approach is often referred to as radiomics, and it is defined as the analysis of high throughput image texture descriptors [10], whereas the DL approach implicitly builds data-driven image descriptors using DL architectures [11]. Both approaches have shown potential for breast percent density classification [12, 13], breast cancer risk assessment [14, 15] and mass detection [16].

We write now: Radiomics is the extraction of quantitative features from medical images, with the aim of correlating them to different health conditions or outcomes [15]. AI-driven radiomics can be divided into models that use classical machine learning algorithms, which use hand-crafted radiomic features [15], and DL approaches, which implicitly build data-driven image descriptors using DL architectures [16]. Both AI-driven approaches have shown potential for breast percent density classification [17, 18], breast cancer risk assessment [19, 20], and mass detection [21]. Recently, AI-supported mammography readings have been suggested to reduce reader workload [22] and improve lesion detection [23].

Line 41-42 While this statement could be true (from a certain point of view) two references are from 2020 and 2018 and the third is a self-citation. What is “often”? There are at least two examples of commercial cancer detection software for mammography that has been shown through randomized clinical trials to decrease workload and increase cancer detection when used in breast cancer screening. There are also several examples of other similar software performing well on external independent validation set.

Thank you for the insightful comment. Indeed, there are commercial cancer detection software for mammography analysis and publications describing models that have good performance in external validation sets. Simultaneously, we argue that limitations in research due to a lack of extensive datasets and model degradation due to domain or distribution shift are common occurrences acknowledged in the literature.

Actions taken: We have modified the wording of the referred section to exclude the uncertainty of the word “often”, and included relevant, recent citations to clarify and illustrate our point better:

We wrote earlier: Although AI-based mammography analysis has demonstrated great potential, these models often fail the test of external validation [26, 27, 28].

We write now: Although AI-based mammography analysis has demonstrated great potential, the lack of access to representative datasets [24, 25, 26, 27] and model degradation due to data distribution shift [28, 29, 30, 31, 32] are commonly reported challenges that are hindering the translation of AI to clinical practice.

Lines 42-45 Are the statements about high heterogeneity meant to be backed up by refs 26-28.

Thank you for pointing this out.

Actions taken: We have corrected the formatting issue.

Lines 49-52 I don’t understand this reasoning. The point of radiomics is to be able to get biomarkers from images and not from biological samples. This seems like circular reasoning to me. We are imaging the biology of the breast, so of course the biology will affect the radiology, how else would anything be detectable? The biological properties of the breast do not affect the textural features, they ARE the textural features (in combination with the radiological imaging technique of course).

Thank you, we agree with this important observation.

Actions taken: We have modified the section for clarity and correctness.

We wrote earlier: Similar to acquisition parameters, the biological properties of the breast affect textural features. Furthermore, as radiomic biomarkers are not initially derived from biological samples but rather from radiological images, the underlying biology affecting radiomic features may also affect the performance of AI models [19].

We write now: On the other hand, the biological properties of the breast, through the interaction with the x-ray radiation, create the radiomic features; the heterogeneity that arises from them is, technically, desired. However, as radiomic biomarkers are not initially derived from biological samples but rather from radiological images, the effect of the interplay between image acquisition and the underlying biology on the performance of AI-based radiomics is unknown [33].

Lines 52-54 What research is scarce? Into the breast micro-tumor environment?

Thank you for pointing out this issue. The sentence should refer to research into how fibroglandular and adipose tissue reflects onto the radiomic features.

Actions taken: We have modified this line for clarity.

We wrote earlier: Research beyond tumor biomarkers into fibroglandular and adipose tissue – from genetic and molecular levels to cellular and tissue levels – is currently scarce but needed to explain how radiomic features may predict the future development of cancer.

We write now: Research into the role of fibroglandular and adipose tissue—from the genetic and molecular levels to cellular and tissue levels—on radiomic features is currently scarce but is needed to explain how radiomic features may predict the future development of cancer.

Lines 56-59 What would be the specific purpose of having non-optimal acquisition performance for validation? Also, degrading image quality to accurately simulate dose reduction is relatively straight-forward to do retrospectively.

Thank you for the insightful point. Rather than a desire to “validate” a model with mammograms acquired with non-optimal parameters, this sentence refers to the fact that retrospective mammography datasets are not acquired in a way that permits the study of the effect of acquisition parameters in computerized analysis or AI applications. We also agree that dose reduction can be simulated post-acquisition in the form of noise, but as we are interested in testing at the radiomic feature level (and then the model level) we consider the rigorousness of acquiring the images with the defined parameters non-negotiable.

Actions taken: We have modified this paragraph for clarity.

We wrote earlier: First, the acquisition parameters in clinical practice used to capture each specific mammogram are optimized for image quality and as a low radiation dose as reasonably achievable: this limits the range of acquisition parameters that are employed on the different breast densities.

We write now: As a source of ionizing radiation, mammographic imaging limits the number of times a person can be imaged. As a result, the acquisition parameters in clinical practice used to capture each specific mammogram are optimized for image quality and for as low a radiation dose as is reasonably achievable: this limits the range of acquisition parameters that are employed on the different breasts, which, in turn, limits the possibilities of analyzing the effect of acquisition parameters on computerized analysis of mammograms derived from in vivo breasts.

Lines 69-72 I think these goals are too non-specific. I understand that it is an exploratory study, but it would benefit from some kind of hypothesis and defined quantitative (or even qualitative) endpoints and figures of merit.

We agree with this observation.

Actions taken: we have made our hypothesis explicit and added necessary details to the objectives.

Our objectives previously read: We aim to systematically study the effect of imaging acquisition parameters and biological factors on computerized mammographic analysis (Figure 1).

1. The first objective is to evaluate how imaging parameters affect the computerized mammographic texture features.

2. The second objective is to evaluate how biological factors across different scales affect computerized mammographic texture features.

Our objectives now read: Our hypothesis posits that *variations in mammography acquisition* *introduce a measurable batch effect.* Under this hypothesis, it is expected that the batch effect significantly affects the performance and generalizability of AI models for breast cancer detection. The characterization of this effect, as well as a better understanding of the link between biological characteristics and mammographic radiomic features, are fundamental to developing robust models (Figure 1). The study has two objectives:

1. Evaluate how imaging parameters affect the computerized mammographic radiomic features by
   - developing a model for the batch effect in mammography images via quantification of its effect in radiomic features; and
   - assessing the impact of acquisition parameters on the performance of AI models for breast cancer detection from mammography.
2. Evaluate how biological factors across different scales affect computerized mammographic features by
   - assessing associations between different biological characteristics of the breast tissue and radiomic features.

METHODS

General comment: I have two general concerns about the Methods and design.

1. What is the role of AI in the project? It is neither in the title nor in the objectives but is mentioned throughout the paper and implied to be important. However, there is no mention in the methods of what kind of AI software or what kind features are going to be tested. You seem to combine AI for cancer detection and for risk assessment, two tasks which are quite different from each other.+

We agree with this observation. AI is an important part of the study, and we will test open-source models found in the literature. As the project includes several levels of analysis of the impact of acquisition parameters (on texture features themselves, on classical machine learning applications, and on DL applications) we have opted to use the term *computerized mammographic analysis.*

Actions taken: We have modified the title and objectives to for clarity on the relevance of computerized analysis.

Our title earlier read: A systematic approach to study the effects of acquisition parameters and biological factors on mammographic texture features using ex-vivo human tissue: a protocol description

Our title now reads: A systematic approach to study the effects of acquisition parameters and biological factors on computerized mammography analysis using ex-vivo human tissue: a protocol description

Our objectives previously read: We aim to systematically study the effect of imaging acquisition parameters and biological factors on computerized mammographic analysis (Figure 1).

1. The first objective is to evaluate how imaging parameters affect the computerized mammographic texture features.

2. The second objective is to evaluate how biological factors across different scales affect computerized mammographic texture features.

Our objectives now read: Our hypothesis posits that *variations in mammography acquisition* *introduce a measurable batch effect.* Under this hypothesis, it is expected that the batch effect significantly affects the performance and generalizability of AI models for breast cancer detection. The characterization of this effect, as well as a better understanding of the link between biological characteristics and mammographic radiomic features, are fundamental to developing robust models (Figure 1). The study has two objectives:

1. Evaluate how imaging parameters affect the computerized mammographic radiomic features by
   - developing a model for the batch effect in mammography images via quantification of its effect in radiomic features; and
   - assessing the impact of acquisition parameters on the performance of AI models for breast cancer detection from mammography.
2. Evaluate how biological factors across different scales affect computerized mammographic features by
   - assessing associations between different biological characteristics of the breast tissue and radiomic features.

2. How are you going to vary acquisition parameters? Is there a set protocol for this with certain increments of tube voltage and mAs? Will this be different for different vendors? How will you compensate for the differences from real clinical images and the mastectomy samples that you will employ? Especially considering that the lack of chest wall attachment will result in a very different level of compression and thickness which will in turn likely make it unsuitable to use the automatic exposure control. Will all breasts be imaged across the same range of setting to provide comparable images?

Thank you for the question. Yes, we have established a protocol in which, by using manual exposure, we set fixed increments to span all the parameter range that we will consider in a machine-specific manner, due to the hardware limitations. The tasks of imaging in- and ex-vivo tissue differ in many aspects, and we have established as many similarities and approximations of the imaging conditions as possible: first, the behavior of resected tissue under compression is one unknown aspect of this study, but the imaging parameters ranges that we will use always include the clinical values that were used for the breast in vivo, and the ranges of parameters used to image all breasts will have significant overlap to make comparison possible; second, while the biological conditions such as tissue oxygenation cannot be replicated in ex-vivo tissue, we will image every sample within hours of its resection, finally, the initial findings of the study on ex-vivo tissue will be validated in an existing screening mammography dataset of more than 300.000 images.

Actions taken: We have modified this paragraph for clarity.

We wrote earlier: Since imaging parameters are a central variable of our study, each breast sample will be imaged at different acquisition parameter settings, yielding several images. The acquisition parameters that will be studied are the compressed breast thickness, compression force, kVp and mAs. As mentioned above, the optimal parameters for image acquisition are dependent on the characteristics of the breast, and the machine used. Our study will stretch these boundaries by virtue of the flexibility that ex-vivo tissue allows. In this way, we will explore a range of parameters that include the clinical range for the given breast sample and reach out to include as much overlap of parameter settings between individual breasts as possible. As there is no available literature on the mammography imaging of ex vivo breast tissue, the specific sets of parameters used will be decided on a breast basis, considering the limitations of each specimen.

We write now: Since imaging parameters are a central variable of our study, each breast sample will be imaged with different combinations of imaging parameters, yielding several images. The acquisition parameters that will be studied are the compressed breast thickness, compression force, kVp, and mAs. We will vary these parameters based on a predefined parameter matrix structured by fixed intervals of kVp and mAs; within each position of this matrix, values of breast thickness and compression force will be used according to the capacity of each specific tissue. For each breast, we will explore a range of parameters covering the clinically used distribution and overlapping with the range of parameters used for all other samples. As there is no available literature on the mammography imaging of ex vivo breast tissue, we will use the clinically used compression force as a guideline, but the specific values used for breast compression force and thickness will be decided on a breast basis, considering the limitations of each specimen.

Lines 151-153 What does it mean that you will “reach out to include as much overlap of parameter settings between individual breast as possible”?

Thank you for the comment. We agree that the sentence was vague. We meant that the different sets of imaging parameters that we will use to image each breast will overlap.

Actions taken: We have modified this paragraph for clarity.

We wrote earlier: Our study will stretch these boundaries by virtue of the flexibility that ex-vivo tissue allows. In this way, we will explore a range of parameters that include the clinical range for the given breast sample and reach out to include as much overlap of parameter settings between individual breasts as possible

We write now: For each breast, we will explore a range of parameters covering the clinically used distribution and overlapping with the range of parameters used for all other samples.

Lines 209-212 While biological-radiological correlations are hailed as the main feature of the study, there is very little detail on the biological and histopathological analysis. What exactly are you going to analyse in the benign adipose and fibroglandular tissue samples? I don’t see how you could e.g. analyse the structural composition of the breast from small tissue samples.

Our study has two equally important goals: evaluating the effect of acquisition parameters on radiomic features and on AI models for mammography analysis, and evaluating how biological factors are reflected in the mammographic features. In this protocol, we detail our approach to achieving our first goal. Specific procedures and analysis for our second goal will be addressed in subsequent works by collaborators performing the tissue analyses.

Actions taken: We have added a clarifying statement to the end of subsection *1.3 Study objectives* as well as complementary information to subsection *2.4.3 Biological and histopathological analysis.* Respectively, they read*:*

This article primarily details the methodology and analysis pertaining to our first objective, with a focus on the study of the acquisition parameters of mammography.

The tissue analyses will include, for example, routine hematoxylin-eosin staining for tissue morphology, label-free second harmonic generation imaging or Masson trichrome staining for collagen in fibroglandular tissue, cytokeratin staining for glandular epithelium, and fluorescent lipid staining for adipocytes. Tissue stainings will be analyzed using microscopic and/or mesoscopic imaging combined with modern digital pathology methods, including AI-driven image analysis.

**Bibliography**

| [1] | S. W. Duffy, L. Tabár, A. Ming-Fang Yen, P. B. B Dean, R. A. Smith, H. Jonsson, S. Törnberg, S. Yueh-Hsia Chiu, S. Li-Sheng Chen, G. Hsiao-Hsuan Jen and et al., "Beneficial effect of consecutive screening mammography examinations on mortality from breast cancer: a prospective study," *Radiology,* p. 299(3):541–547, 2021. |
| --- | --- |
| [2] | S. Mayor, "Survival of women treated for early breast cancer detected by screening is same as in general population, audit shows," *BMJ: British Medical Journal,* vol. 336, no. 7658, p. 1398, 2008. |
| [3] | C. Allemani, T. Matsuda, V. Di Carlo, R. Harewood, M. Matz, M. Nikšić, A. Bonaventure, M. Valkov, C. J. Johnson, J. Estève, O. J. Ogunbiyi and G. Azevedo e Silva, "Global surveillance of trends in cancer survival 2000–14 (CONCORD-3): analysis of individual records for 37 513 025 patients diagnosed with one of 18 cancers from 322 population-based registries in 71 countries," *The Lancet,* vol. 391, no. 10125, pp. 1023 - 1075, 2018. |
| [4] | H. Sung, J. Ferlay, R. L. Siegel, M. Laversanne, I. Soerjomataram, A. Jemal and F. Bray, "Global cancer statistics 2020: Globocan estimates of incidence and mortality worldwide for 36 cancers in 185 countries," *CA: a cancer journal for clinicians,* p. 71(3):209–249, 2021. |
| [5] | L. Donata, D. Silvia, F. Crystal, L. A. Jesus, N. Luciana, N. Ciaran, N. Nicholas, U. Asli and V. F. Silvia, "Report of a European survey on the organisation of breast cancer care services - Supporting information for the European Commission initiative on breast cancer," Publications Office of the European Union, Luxembourg (Luxembourg), 2014. |
| [6] | L. Tabár, V. Bedrich , T. Hsiu-Hsi Chen, A. Ming-Fang Yen, A. Cohen, T. Tot, S. Yueh-Hsia Chiu, S. Li-Sheng Chen, J. Ching-Yuan Fann, J. Rosell and et al., "Swedish two-county trial: impact of mammographic screening on breast cancer mortality during 3 decades," *Radiology,* p. 260(3):658–663, 2011. |
| [7] | M. G. Marmot, D. G. Altman, D. A. Cameron, J. A. Dewar, S. G. Thompson, M. Wilcox and The Independent UKPanel on Breast Cancer Screening, "The benefits and harms of breast cancer screening: an independent review," *The Lancet,* vol. 380, no. 9855, pp. 1778-1786, 2012. |
| [8] | D. Stamatia, L. Johnston, R. Highnam, A. Arieno, R. Morgan and A. Chan, "Using volumetric breast density to quantify the potential masking risk of mammographic density," *AJR Am J Roentgenol,* vol. 208, no. 1, pp. 222--227, 2017. |
| [9] | H. Hussein, E. Abbas, S. Keshavarzi, R. Fazelzad, K. Bukhanov, S. Kulkarni, F. Au, S. Ghai, A. Alabousi and . V. Freitas, "Supplemental Breast Cancer Screening in Women with Dense Breasts and Negative Mammography: A Systematic Review and Meta-Analysis," *Radiology,* vol. 306(3), p. e221785, 2023. |
| [10] | P. B. Lam, P. M. Vacek, B. M. Geller and H. B. Muss, "The association of increased weight, body mass index, and tissue density with the risk of breast carcinoma in Vermont," *Cancer: Interdisciplinary International Journal of the American Cancer Society,* vol. 89, no. 2, pp. 369--375, 2000. |
| [11] | N. F. Boyd, G. A. Lockwood, J. W. Byng, D. L. Tritchler and M. J. Yaffe, "Mammographic densities and breast cancer risk," *Cancer epidemiology, biomarkers & prevention: a publication of the American Association for Cancer Research, cosponsored by the American Society of Preventive Oncology,* vol. 7, no. 12, pp. 1133--1144, 1998. |
| [12] | R. M. Mann, A. Athanasiou, P. A. Baltzer, J. Camps-Herrero, P. Clauser, E. M. Fallenberg, G. Forrai, M. H. Fuchsjäger, T. H. Helbich, F. Killburn-Toppin and others, "Breast cancer screening in women with extremely dense breasts recommendations of the European Society of Breast Imaging (EUSOBI)," *European radiology,* vol. 32, no. 6, pp. 4036--4045, 2022. |
| [13] | N. F. Boyd, G. A. Lockwood, J. W. Byng, D. L. Tritchler and M. J. Yaffe, "Mammographic densities and breast cancer risk," *Cancer epidemiology, biomarkers \& prevention: a publication of the American Association for Cancer Research, cosponsored by the American Society of Preventive Oncology,* vol. 12, no. 7, pp. 1133--1144, 1998. |
| [14] | V. A. McCormack and I. dos Santos Silva, "Breast density and parenchymal patterns as markers of breast cancer risk: a meta-analysis," *Cancer Epidemiol Biomarkers Prev,* vol. 15(6), pp. 1159-69, 2006. |
| [15] | M. E. Mayerhoefer, A. Materka, G. Langs, I. Häggström, P. Szczypiński, P. Gibbs and G. Cook, "Introduction to radiomics," *Journal of Nuclear Medicine,* vol. 61, no. 4, pp. 488--495, 2020. |
| [16] | A. Bhowmik and S. Eskreis-Winkler, "Deep learning in breast imaging," *BJR\| Open,* vol. 4, p. 20210060, 2022. |
| [17] | G. Liasis, C. Pattichis and S. Petroudi, "Combination of different texture features for mammographic breast density classification," in *2012 IEEE 12th International Conference on Bioinformatics \& Bioengineering (BIBE)*, Larnaca, Cyprus, 2012. |
| [18] | C. Lehman, A. Yala, T. Schuster, B. Dontchos, M. Bahl, K. Swanson and R. Barzilay, "Mammographic breast density assessment using deep learning: clinical implementation," *Radiology,* vol. 290, pp. 52-58, 2019. |
| [19] | S. Pertuz, A. Sassi, K. Holli-Helenius, J. Kääräinen, I. Rinta-Kiikka, A.-L. Lääperi and O. Arponen, "Clinical evaluation of a fully-automated parenchymal analysis software for breast cancer risk assessment: A pilot study in a Finnish sample," *European journal of radiology,* vol. 121, p. 108710, 2019. |
| [20] | H. Li, M. Giger, B. Q. Huynh and N. O. Antropova, "Deep learning in breast cancer risk assessment: evaluation of convolutional neural networks on a clinical dataset of full-field digital mammograms," *Journal of medical imaging,* vol. 4, pp. 041304--041304, 2017. |
| [21] | A. Rodriguez-Ruiz, K. Lång, A. Gubern-Merida, M. Broeders, G. Gennaro, P. Clauser, T. H. Helbich, M. Chevalier, T. Tan, T. Mertelmeier and et al, "Stand-alone artificial intelligence for breast cancer detection in mammography: comparison with 101 radiologists," *JNCI: Journal of the National Cancer Institute,* vol. 111(9), p. 916–922, 2019. |
| [22] | T. Kyono, . F. J. Gilbert and M. van der Schaar, "Improving Workflow Efficiency for Mammography Using Machine Learning," *Journal of the American College of Radiology,* vol. 17, no. 1, pp. 56-63, 2020. |
| [23] | Y. Sun, Y. Qu, D. Wang, Y. Li, L. Ye, . J. Du, B. Xu, B. Li, X. Li, K. Zhang, Y. Shi, R. Sun, Y. Wang, R. Long, . D. Chen, H. Li, L. Wang and M. Cao, "Deep learning model improves radiologists’ performance in detection and classification of breast lesions," *Chinese Journal of Cancer Research,* vol. 33, no. 6, p. 682–693, 2021 . |
| [24] | D. J. Wong, Z. Gandomkar, W.-J. Wu, G. Zhang, W. Gao, X. He, Y. Wang and W. Reed, "Artificial intelligence and convolution neural networks assessing mammographic images: A narrative literature review," *Journal of medical radiation sciences,* vol. 67, no. 2, pp. 134--142, 2020. |
| [25] | A. W. Anderson, M. L. Marinovich, N. Houssami, K. P. Lowry, J. G. Elmore, D. S. Buist, S. Hofvind and C. I. Lee, "Independent external validation of artificial intelligence algorithms for automated interpretation of screening mammography: a systematic review," *Journal of the American College of Radiology,* vol. 19, no. 2, pp. 259--273, 2022. |
| [26] | M. B. Ammar, F. L. Ayechi, R. Ksantini and H. Mahjoubi, "Pre-Trained Deep Convolutional Neural Network Architectures for Breast Cancer Diagnosis in Mammography: Current State-Of-The-Art," in *2023 International Conference on Innovations in Intelligent Systems and Applications (INISTA)*, 2023. |
| [27] | W. C. Ou, D. Polat and B. E. Dogan, "Deep learning in breast radiology: current progress and future directions," *European Radiology,* vol. 31, pp. 4872--4885, 2021. |
| [28] | X. Wang, G. Liang, Y. Zhang, H. Blanton, Z. Bessinger and N. Jacobs, "Inconsistent performance of deep learning models on mammogram classification," *Journal of the American College of Radiology,* vol. 17, pp. 796--803, 2020. |
| [29] | C. F. de Vries, S. J. Colosimo, R. T. Staff, J. A. Dymiter, J. Yearsley, D. Dinneen, M. Boyle, D. J. Harrison, L. A. Anderson, G. Lip and others, "Impact of different mammography systems on artificial intelligence performance in breast cancer screening," *Radiology: Artificial Intelligence,* vol. 5, no. 3, p. e220146, 2023. |
| [30] | S. Pertuz, A. Sassi, O. Arponen, K. Holli-Helenius, A.-L. Lääperi and I. Rinta-Kiikka, "Do mammographic systems affect the performance of computerized parenchymal analysis?," in *2019 41st Annual International Conference of the IEEE Engineering in Medicine and Biology Society (EMBC)*, Berlin, Germany, 2019 . |
| [31] | W. Hsu, D. S. Hippe, N. Nakhaei, P.-C. Wang, B. Zhu, N. Siu, M. E. Ahsen, W. Lotter, A. G. Sorensen, A. Naeim and others, "External validation of an ensemble model for automated mammography interpretation by artificial intelligence," *JAMA network open,* vol. 5, no. 11, pp. e2242343--e2242343, 2022. |
| [32] | J. J. Condon, V. Trinh, K. A. Hall, M. Reintals, A. S. Holmes, L. Oakden-Rayner and L. J. Palmer, "Impact of transfer learning using local data on performance of a deep learning model for screening mammography," *Radiology: Artificial Intelligence,* vol. 6, no. 4, p. e230383, 2024. |
| [33] | M. R. Tomaszewski and R. J. Gillies, "The biological meaning of radiomic features," *Radiology,* vol. 298, pp. 505--516, 2021. |
